# 5-Bromo-2,7-dimethyl-3-(3-methyl­phenyl­sulfon­yl)-1-benzo­furan

**DOI:** 10.1107/S1600536814008149

**Published:** 2014-04-16

**Authors:** Hong Dae Choi, Pil Ja Seo, Uk Lee

**Affiliations:** a Department of Chemistry, Dongeui University, San 24 Kaya-dong, Busanjin-gu, Busan 614-714, Republic of Korea; b Department of Chemistry, Pukyong National University, 599-1 Daeyeon 3-dong, Nam-gu, Busan 608-737, Republic of Korea

## Abstract

In the title compound, C_17_H_15_BrO_3_S, the dihedral angle between the mean planes of the benzo­furan and 3-methyl­phenyl rings is 77.37 (5)°. In the crystal, mol­ecules are linked *via* pairs of Br⋯O [Br⋯O = 3.335 (2) Å] contacts into inversion dimers. These dimers are further linked by C—H⋯O hydrogen bonds and π–π inter­actions between the benzene and furan rings of neighbouring mol­ecules [centroid–centroid separation = 3.884 (3) Å] into supra­molecular chains running along the *a*-axis direction.

## Related literature   

For background information and the crystal structures of related compounds, see: Choi *et al.* (2011 ▶, 2012 ▶, 2013 ▶). For a review of halogen bonding, see: Politzer *et al.* (2007 ▶).
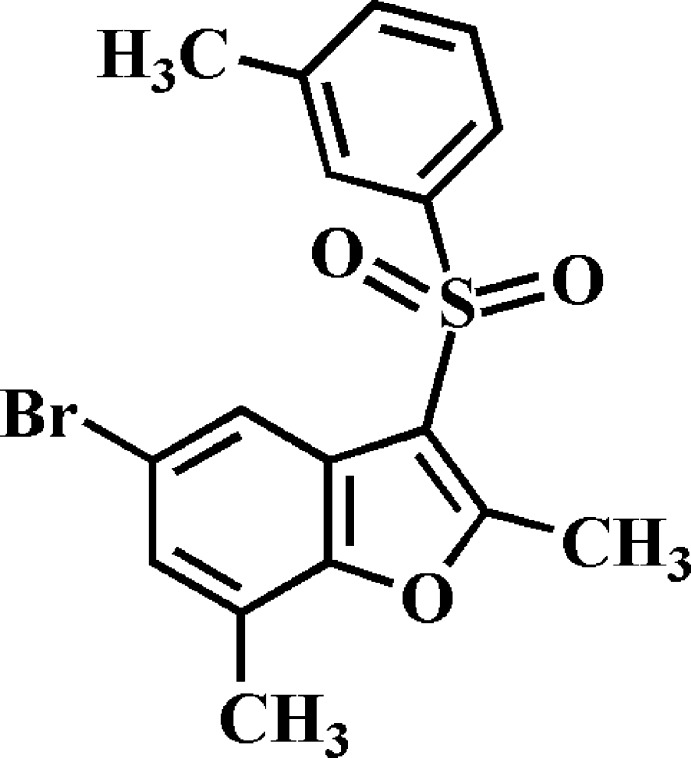


## Experimental   

### 

#### Crystal data  

C_17_H_15_BrO_3_S
*M* *_r_* = 379.26
Monoclinic, 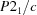

*a* = 8.8813 (3) Å
*b* = 6.5976 (2) Å
*c* = 26.5044 (8) Å
β = 97.635 (1)°
*V* = 1539.26 (8) Å^3^
*Z* = 4
Mo *K*α radiation
μ = 2.82 mm^−1^
*T* = 296 K
0.50 × 0.46 × 0.15 mm

#### Data collection  

Bruker SMART APEXII CCD diffractometer
Absorption correction: multi-scan (*SADABS*; Bruker, 2009 ▶) *T*_min_ = 0.379, *T*_max_ = 0.746
26392 measured reflections
3831 independent reflections
3188 reflections with *I* > 2σ(*I*)
*R*_int_ = 0.043

#### Refinement  

*R*[*F* ^2^ > 2σ(*F* ^2^)] = 0.031
*wR*(*F* ^2^) = 0.081
*S* = 1.07
3831 reflections
202 parameters
H-atom parameters constrained
Δρ_max_ = 0.56 e Å^−3^
Δρ_min_ = −0.45 e Å^−3^

### 

Data collection: *APEX2* (Bruker, 2009 ▶); cell refinement: *SAINT* (Bruker, 2009 ▶); data reduction: *SAINT*; program(s) used to solve structure: *SHELXS97* (Sheldrick, 2008 ▶); program(s) used to refine structure: *SHELXL97* (Sheldrick, 2008 ▶); molecular graphics: *ORTEP-3 for Windows* (Farrugia, 2012 ▶) and *DIAMOND* (Brandenburg, 1998 ▶); software used to prepare material for publication: *SHELXL97*.

## Supplementary Material

Crystal structure: contains datablock(s) I. DOI: 10.1107/S1600536814008149/bh2497sup1.cif

Structure factors: contains datablock(s) I. DOI: 10.1107/S1600536814008149/bh2497Isup2.hkl

Supporting information file. DOI: 10.1107/S1600536814008149/bh2497Isup3.cml

CCDC reference: 996711

Additional supporting information:  crystallographic information; 3D view; checkCIF report

## Figures and Tables

**Table 1:** Hydrogen-bond geometry (Å, °)

*D*—H⋯*A*	*D*—H	H⋯*A*	*D*⋯*A*	*D*—H⋯*A*
C10—H10*B*⋯O2^i^	0.96	2.54	3.338 (3)	141
C17—H17*C*⋯O3^ii^	0.96	2.41	3.357 (3)	170

Symmetry codes: (i) 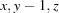; (ii) 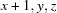.

**Fig. 1.**
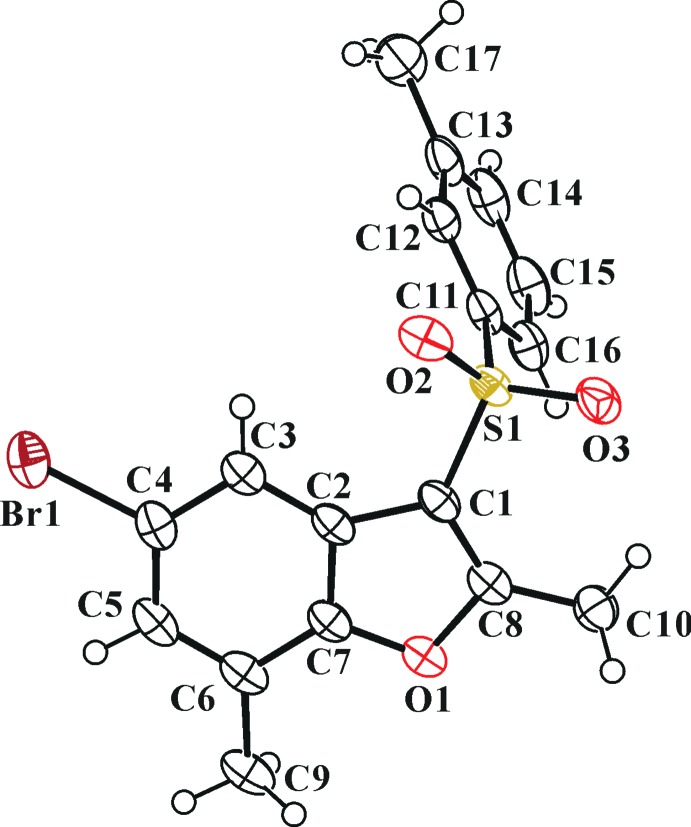
The molecular structure of the title molecule with the atom numbering scheme The displacement ellipsoids are drawn at the 50% probability level. The hydrogen atoms are presented as small spheres of arbitrary radius.

**Fig. 2.**
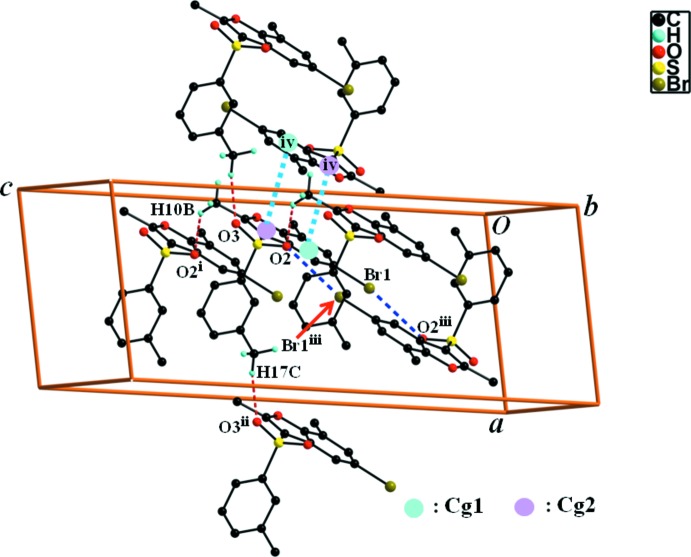
A view of the C—H···O, Br···O and π···π interactions (dotted lines) in the crystal structure of the title compound. H atoms non-participating in hydrogen-bonding were omitted for clarity. [Symmetry codes: (i) x, y - 1, z; (ii) x + 1, y, z; (iii) - x + 1, - y + 2, - z + 1; (iv) - x, - y + 1, - z + 1.]

## supplementary crystallographic information

### 2.1. Synthesis and crystallization

3-Chloro­per­oxy­benzoic acid (77%, 448 mg, 2.0 mmol) was added in small portions to a stirred solution of 5-bromo-2,7-di­methyl-3-(3-methyl­phenyl­sulfanyl)-1-benzo­furan (312 mg, 0.9 mmol) in di­chloro­methane (40 mL) at 273 K. After being stirred at room temperature for 8 h., the mixture was washed with saturated sodium bicarbonate solution and the organic layer was separated, dried over magnesium sulfate, filtered and concentrated at reduced pressure. The residue was purified by column chromatography (hexane–ethyl acetate, 4:1 *v*/*v*) to afford the title compound as a colorless solid [yield 68%, m.p. 438–439 K; *R*_f_ = 0.51 (hexane–ethyl acetate, 4:1 *v*/*v*)]. Single crystals suitable for X-ray diffraction were prepared by slow evaporation of a solution of the title compound in ethyl acetate at room temperature.

### 2.2. Refinement

Crystal data, data collection and structure refinement details are summarized in Table 1. All H atoms were positioned geometrically and refined using a riding model, with C—H = 0.95 Å for aryl and 0.98 Å for methyl H atoms, respectively. *U*_iso_ (H) = 1.2*U*_eq_ (C) for aryl and 1.5*U*_eq_ (C) for methyl H atoms. The positions of methyl hydrogens were optimized using the SHELXL-97's command AFIX 137 (Sheldrick, 2008).

### 3. Results and discussion

As a part of our ongoing study of 5-bromo-2,7-di­methyl-1-benzo­furan derivatives containing cyclo­hexyl­sulfonyl (Choi *et al.*, 2011), 4-fluoro­phenyl­sulfonyl (Choi *et al.*, 2012) and 4-methyl­phenyl­sulfinyl (Choi *et al.*, 2013) substituents in the 3-position, we report here on the crystal structure of the title compound.

In the title molecule (Fig. 1), the benzo­furan ring system is essentially planar, with a mean deviation of 0.010 (2) Å from the least-squares plane defined by the nine constituent atoms. The 3-methyl­phenyl ring is essentially planar, with a mean deviation of 0.004 (1) Å from the least-squares plane defined by the six constituent atoms. The dihedral angle formed by the benzo­furan ring system and the 3-methyl­phenyl ring is 77.37 (5)°. In the crystal structure (Fig. 2), molecules are linked by pairs of Br···O halogen-bondings (Politzer *et al.* 2007) between the bromine and the O atom of the O═S═O unit [Br1···O2^iii^ = 3.335 (2) Å, C4—Br1···O2^iii^ = 168.67 (6)°, symmetry code: (iii) -*x*+1, -*y*+2, -*z*+1]. These dimers are further linked by C—H···O hydrogen bonds (Table 1), and by π···π inter­actions between the benzene and furan rings of neighbouring molecules, with a Cg1···Cg2^iv^ distance of 3.884 (3) Å and an inter­planar distance of 3.440 (3) Å resulting in a slippage of 1.804 (3) Å (Cg1 and Cg2 are the centroids of the C2–C7 benzene ring and the C1/C2/C7/O1/C8 furan ring, respectively), forming supra­molecular chains running along the *a*–axis direction.

### Crystal data

**Table:**

C_17_H_15_BrO_3_S	*F*(000) = 768
*M**_r_* = 379.26	*D*_x_ = 1.637 Mg m^−^^3^
Monoclinic, *P*2_1_/*c*	Melting point = 439–438 K
Hall symbol: -P 2ybc	Mo *K*α radiation, λ = 0.71073 Å
*a* = 8.8813 (3) Å	Cell parameters from 9774 reflections
*b* = 6.5976 (2) Å	θ = 2.3–28.2°
*c* = 26.5044 (8) Å	µ = 2.82 mm^−^^1^
β = 97.635 (1)°	*T* = 296 K
*V* = 1539.26 (8) Å^3^	Block, colourless
*Z* = 4	0.50 × 0.46 × 0.15 mm

### Data collection

**Table:**

Bruker SMART APEXII CCD diffractometer	3831 independent reflections
Radiation source: rotating anode	3188 reflections with *I* > 2σ(*I*)
Graphite multilayer monochromator	*R*_int_ = 0.043
Detector resolution: 10.0 pixels mm^-1^	θ_max_ = 28.3°, θ_min_ = 1.6°
φ and ω scans	*h* = −11→11
Absorption correction: multi-scan (*SADABS*; Bruker, 2009)	*k* = −8→8
*T*_min_ = 0.379, *T*_max_ = 0.746	*l* = −35→35
26392 measured reflections	

### Refinement

**Table:**

Refinement on *F*^2^	Primary atom site location: structure-invariant direct methods
Least-squares matrix: full	Secondary atom site location: difference Fourier map
*R*[*F*^2^ > 2σ(*F*^2^)] = 0.031	Hydrogen site location: difference Fourier map
*wR*(*F*^2^) = 0.081	H-atom parameters constrained
*S* = 1.07	*w* = 1/[σ^2^(*F*_o_^2^) + (0.0364*P*)^2^ + 0.7777*P*] where *P* = (*F*_o_^2^ + 2*F*_c_^2^)/3
3831 reflections	(Δ/σ)_max_ = 0.002
202 parameters	Δρ_max_ = 0.56 e Å^−^^3^
0 restraints	Δρ_min_ = −0.45 e Å^−^^3^

### Fractional atomic coordinates and isotropic or equivalent isotropic displacement parameters (Å^2^)

**Table:**

	*x*	*y*	*z*	*U*_iso_*/*U*_eq_	
Br1	0.46068 (3)	0.80654 (4)	0.428686 (8)	0.04118 (9)	
S1	0.26202 (5)	0.72154 (8)	0.641971 (18)	0.02783 (11)	
O1	0.09711 (16)	0.2852 (2)	0.55049 (5)	0.0314 (3)	
O2	0.27849 (16)	0.9169 (2)	0.61959 (5)	0.0352 (3)	
O3	0.16243 (16)	0.7009 (3)	0.68033 (6)	0.0380 (4)	
C1	0.2041 (2)	0.5514 (3)	0.59334 (7)	0.0284 (4)	
C2	0.2454 (2)	0.5613 (3)	0.54261 (7)	0.0277 (4)	
C3	0.3313 (2)	0.6908 (3)	0.51640 (7)	0.0305 (4)	
H3	0.3777	0.8062	0.5314	0.037*	
C4	0.3435 (2)	0.6375 (4)	0.46673 (7)	0.0318 (4)	
C5	0.2756 (2)	0.4660 (4)	0.44286 (7)	0.0347 (5)	
H5	0.2888	0.4380	0.4093	0.042*	
C6	0.1884 (2)	0.3360 (3)	0.46849 (8)	0.0325 (4)	
C7	0.1763 (2)	0.3932 (3)	0.51806 (7)	0.0291 (4)	
C8	0.1157 (2)	0.3850 (3)	0.59611 (7)	0.0302 (4)	
C9	0.1153 (3)	0.1474 (4)	0.44553 (8)	0.0415 (5)	
H9A	0.1709	0.0312	0.4596	0.062*	
H9B	0.1157	0.1499	0.4093	0.062*	
H9C	0.0125	0.1399	0.4529	0.062*	
C10	0.0370 (3)	0.2911 (3)	0.63612 (9)	0.0368 (5)	
H10A	0.0463	0.3777	0.6655	0.055*	
H10B	0.0821	0.1619	0.6454	0.055*	
H10C	−0.0685	0.2730	0.6235	0.055*	
C11	0.4444 (2)	0.6401 (3)	0.66941 (7)	0.0283 (4)	
C12	0.5609 (2)	0.7803 (3)	0.67428 (7)	0.0314 (4)	
H12	0.5452	0.9090	0.6603	0.038*	
C13	0.7026 (2)	0.7273 (4)	0.70039 (8)	0.0389 (5)	
C14	0.7213 (3)	0.5329 (4)	0.71966 (8)	0.0461 (6)	
H14	0.8152	0.4950	0.7369	0.055*	
C15	0.6045 (3)	0.3937 (4)	0.71410 (8)	0.0444 (6)	
H15	0.6208	0.2638	0.7274	0.053*	
C16	0.4631 (2)	0.4458 (4)	0.68888 (8)	0.0367 (5)	
H16	0.3834	0.3533	0.6852	0.044*	
C17	0.8290 (2)	0.8819 (5)	0.70728 (10)	0.0535 (7)	
H17A	0.8362	0.9388	0.7409	0.080*	
H17B	0.8078	0.9877	0.6825	0.080*	
H17C	0.9233	0.8176	0.7029	0.080*	

### Atomic displacement parameters (Å^2^)

**Table:**

	*U*^11^	*U*^22^	*U*^33^	*U*^12^	*U*^13^	*U*^23^
Br1	0.04301 (14)	0.04977 (17)	0.03230 (13)	0.01099 (10)	0.01067 (9)	0.00424 (9)
S1	0.0264 (2)	0.0325 (3)	0.0236 (2)	0.00823 (19)	−0.00028 (17)	−0.00524 (18)
O1	0.0345 (7)	0.0306 (8)	0.0276 (7)	0.0047 (6)	−0.0014 (6)	−0.0037 (6)
O2	0.0379 (7)	0.0294 (8)	0.0352 (8)	0.0090 (6)	−0.0061 (6)	−0.0033 (6)
O3	0.0309 (7)	0.0511 (11)	0.0329 (8)	0.0049 (7)	0.0072 (6)	−0.0131 (7)
C1	0.0293 (9)	0.0319 (11)	0.0229 (9)	0.0079 (8)	−0.0007 (7)	−0.0030 (7)
C2	0.0272 (9)	0.0315 (11)	0.0228 (9)	0.0105 (8)	−0.0028 (7)	−0.0035 (7)
C3	0.0295 (9)	0.0333 (12)	0.0276 (9)	0.0086 (8)	−0.0002 (7)	−0.0019 (8)
C4	0.0314 (9)	0.0382 (12)	0.0255 (9)	0.0132 (8)	0.0027 (7)	0.0025 (8)
C5	0.0359 (10)	0.0439 (13)	0.0230 (9)	0.0166 (9)	−0.0016 (8)	−0.0055 (8)
C6	0.0328 (10)	0.0336 (12)	0.0288 (10)	0.0137 (8)	−0.0049 (8)	−0.0061 (8)
C7	0.0295 (9)	0.0304 (11)	0.0253 (9)	0.0100 (8)	−0.0033 (7)	−0.0012 (8)
C8	0.0317 (9)	0.0304 (11)	0.0271 (9)	0.0100 (8)	−0.0012 (7)	−0.0028 (8)
C9	0.0484 (12)	0.0394 (13)	0.0332 (11)	0.0111 (10)	−0.0072 (9)	−0.0118 (9)
C10	0.0409 (11)	0.0353 (13)	0.0343 (11)	0.0029 (9)	0.0052 (9)	−0.0013 (9)
C11	0.0283 (9)	0.0390 (11)	0.0174 (8)	0.0115 (8)	0.0025 (7)	−0.0011 (7)
C12	0.0287 (9)	0.0419 (13)	0.0239 (9)	0.0067 (8)	0.0044 (7)	−0.0019 (8)
C13	0.0285 (10)	0.0651 (16)	0.0235 (9)	0.0108 (10)	0.0057 (8)	−0.0055 (10)
C14	0.0348 (11)	0.0774 (19)	0.0253 (10)	0.0247 (12)	0.0005 (8)	0.0031 (11)
C15	0.0542 (13)	0.0508 (15)	0.0275 (10)	0.0248 (12)	0.0033 (10)	0.0101 (10)
C16	0.0405 (11)	0.0430 (13)	0.0266 (9)	0.0113 (9)	0.0050 (8)	0.0036 (9)
C17	0.0263 (10)	0.086 (2)	0.0482 (14)	0.0016 (12)	0.0042 (9)	−0.0118 (14)

### Geometric parameters (Å, º)

**Table:**

Br1—C4	1.903 (2)	C9—H9A	0.9600
Br1—O2^i^	3.3350 (16)	C9—H9B	0.9600
S1—O2	1.4341 (17)	C9—H9C	0.9600
S1—O3	1.4400 (15)	C10—H10A	0.9600
S1—C1	1.735 (2)	C10—H10B	0.9600
S1—C11	1.7687 (19)	C10—H10C	0.9600
O1—C8	1.368 (2)	C11—C12	1.381 (3)
O1—C7	1.379 (3)	C11—C16	1.383 (3)
C1—C8	1.358 (3)	C12—C13	1.398 (3)
C1—C2	1.441 (3)	C12—H12	0.9300
C2—C7	1.387 (3)	C13—C14	1.382 (4)
C2—C3	1.391 (3)	C13—C17	1.510 (4)
C3—C4	1.381 (3)	C14—C15	1.379 (4)
C3—H3	0.9300	C14—H14	0.9300
C4—C5	1.394 (3)	C15—C16	1.385 (3)
C5—C6	1.392 (3)	C15—H15	0.9300
C5—H5	0.9300	C16—H16	0.9300
C6—C7	1.385 (3)	C17—H17A	0.9600
C6—C9	1.495 (3)	C17—H17B	0.9600
C8—C10	1.481 (3)	C17—H17C	0.9600
			
C4—Br1—O2^i^	168.67 (6)	H9A—C9—H9B	109.5
O2—S1—O3	118.89 (9)	C6—C9—H9C	109.5
O2—S1—C1	108.10 (9)	H9A—C9—H9C	109.5
O3—S1—C1	108.30 (10)	H9B—C9—H9C	109.5
O2—S1—C11	107.76 (10)	C8—C10—H10A	109.5
O3—S1—C11	107.20 (9)	C8—C10—H10B	109.5
C1—S1—C11	105.88 (9)	H10A—C10—H10B	109.5
C8—O1—C7	106.91 (16)	C8—C10—H10C	109.5
C8—C1—C2	107.93 (17)	H10A—C10—H10C	109.5
C8—C1—S1	126.84 (15)	H10B—C10—H10C	109.5
C2—C1—S1	125.22 (16)	C12—C11—C16	122.29 (19)
C7—C2—C3	119.64 (18)	C12—C11—S1	117.95 (16)
C7—C2—C1	104.47 (18)	C16—C11—S1	119.54 (17)
C3—C2—C1	135.88 (19)	C11—C12—C13	119.5 (2)
C4—C3—C2	115.9 (2)	C11—C12—H12	120.2
C4—C3—H3	122.0	C13—C12—H12	120.2
C2—C3—H3	122.0	C14—C13—C12	118.1 (2)
C3—C4—C5	123.7 (2)	C14—C13—C17	122.1 (2)
C3—C4—Br1	118.26 (17)	C12—C13—C17	119.8 (2)
C5—C4—Br1	117.99 (15)	C15—C14—C13	121.8 (2)
C6—C5—C4	120.94 (18)	C15—C14—H14	119.1
C6—C5—H5	119.5	C13—C14—H14	119.1
C4—C5—H5	119.5	C14—C15—C16	120.5 (2)
C7—C6—C5	114.4 (2)	C14—C15—H15	119.8
C7—C6—C9	122.0 (2)	C16—C15—H15	119.8
C5—C6—C9	123.63 (19)	C11—C16—C15	117.8 (2)
O1—C7—C6	124.08 (19)	C11—C16—H16	121.1
O1—C7—C2	110.57 (16)	C15—C16—H16	121.1
C6—C7—C2	125.3 (2)	C13—C17—H17A	109.5
C1—C8—O1	110.12 (17)	C13—C17—H17B	109.5
C1—C8—C10	135.10 (19)	H17A—C17—H17B	109.5
O1—C8—C10	114.78 (19)	C13—C17—H17C	109.5
C6—C9—H9A	109.5	H17A—C17—H17C	109.5
C6—C9—H9B	109.5	H17B—C17—H17C	109.5
			
O2—S1—C1—C8	−149.32 (17)	C3—C2—C7—O1	179.96 (16)
O3—S1—C1—C8	−19.3 (2)	C1—C2—C7—O1	0.1 (2)
C11—S1—C1—C8	95.42 (18)	C3—C2—C7—C6	2.0 (3)
O2—S1—C1—C2	32.05 (18)	C1—C2—C7—C6	−177.80 (18)
O3—S1—C1—C2	162.10 (16)	C2—C1—C8—O1	0.2 (2)
C11—S1—C1—C2	−83.20 (18)	S1—C1—C8—O1	−178.57 (14)
C8—C1—C2—C7	−0.2 (2)	C2—C1—C8—C10	−179.5 (2)
S1—C1—C2—C7	178.62 (14)	S1—C1—C8—C10	1.7 (3)
C8—C1—C2—C3	180.0 (2)	C7—O1—C8—C1	−0.2 (2)
S1—C1—C2—C3	−1.2 (3)	C7—O1—C8—C10	179.60 (16)
C7—C2—C3—C4	−1.2 (3)	O2—S1—C11—C12	12.15 (17)
C1—C2—C3—C4	178.6 (2)	O3—S1—C11—C12	−116.91 (15)
C2—C3—C4—C5	0.1 (3)	C1—S1—C11—C12	127.64 (16)
C2—C3—C4—Br1	−179.53 (13)	O2—S1—C11—C16	−173.10 (15)
O2^i^—Br1—C4—C3	58.1 (4)	O3—S1—C11—C16	57.84 (18)
O2^i^—Br1—C4—C5	−121.5 (3)	C1—S1—C11—C16	−57.62 (18)
C3—C4—C5—C6	0.4 (3)	C16—C11—C12—C13	−1.1 (3)
Br1—C4—C5—C6	−179.97 (15)	S1—C11—C12—C13	173.54 (15)
C4—C5—C6—C7	0.2 (3)	C11—C12—C13—C14	1.2 (3)
C4—C5—C6—C9	−178.61 (19)	C11—C12—C13—C17	−178.06 (19)
C8—O1—C7—C6	177.97 (18)	C12—C13—C14—C15	−0.6 (3)
C8—O1—C7—C2	0.0 (2)	C17—C13—C14—C15	178.7 (2)
C5—C6—C7—O1	−179.12 (17)	C13—C14—C15—C16	−0.3 (3)
C9—C6—C7—O1	−0.3 (3)	C12—C11—C16—C15	0.2 (3)
C5—C6—C7—C2	−1.5 (3)	S1—C11—C16—C15	−174.34 (15)
C9—C6—C7—C2	177.39 (19)	C14—C15—C16—C11	0.5 (3)

Symmetry code: (i) −*x*+1, −*y*+2, −*z*+1.

### Hydrogen-bond geometry (Å, º)

**Table:**

*D*—H···*A*	*D*—H	H···*A*	*D*···*A*	*D*—H···*A*
C10—H10*B*···O2^ii^	0.96	2.54	3.338 (3)	141
C17—H17*C*···O3^iii^	0.96	2.41	3.357 (3)	170

Symmetry codes: (ii) *x*, *y*−1, *z*; (iii) *x*+1, *y*, *z*.
